# Ablation of AVNRT in a Setting of Slow Pathway–Dominant Conduction

**DOI:** 10.1016/j.jaccas.2025.106059

**Published:** 2025-12-03

**Authors:** Liuguang Song, Simin Cai, Jian Ye, Yaping Wang

**Affiliations:** Department of Cardiology, The Second Affiliated Hospital, School of Medicine, Zhejiang University, Hangzhou, China

**Keywords:** dual atrioventricular nodal pathways, narrow QRS tachycardia, radiofrequency ablation, slow pathway dominant conduction, supraventricular tachycardia

## Abstract

**Background:**

We report a case of paroxysmal supraventricular tachycardia with slow pathway–dominant conduction and prolonged PR interval in sinus rhythm.

**Case Summary:**

A 33-year-old man presented with persistent palpitations and chest tightness, with narrow QRS tachycardia with a prolonged PR interval on electrocardiogram. After the administration of isoproterenol, the PR interval normalized. During the electrophysiology study, atrioventricular node conduction jumping, echoes, and dual ventricular responses were observed. After ablation, the fast pathway function of the atrioventricular node was restored, and electrocardiography revealed a normal PR interval.

**Discussion:**

This case demonstrates that slow pathway–dominant atrioventricular nodal re-entrant tachycardia can be successfully treated by slow pathway ablation, while ensuring normal fast pathway function is key to avoiding atrioventricular block.

**Take-Home Message:**

Slow pathway ablation effectively treats this arrhythmia, and the preservation of fast pathway function is crucial to avoid atrioventricular block.

## Visual Sumamry

**Figure:**
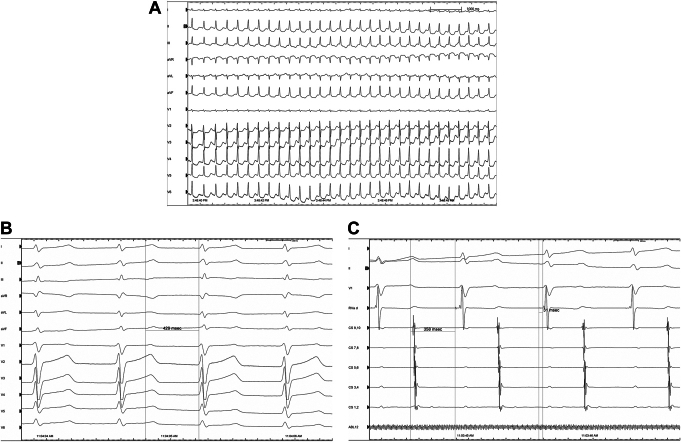

## History of Presentation

A 33-year-old man presented to the Department of Cardiology at the Second Affiliated Hospital of Zhejiang University School of Medicine with apparent recurrent palpitations for over 2 years and aggravation for 3 days. The palpitations occurred without obvious precipitant, began and ended abruptly, and were associated with chest tightness during episodes. The patient was hospitalized in our department when the symptoms aggravated to persistent palpitations and chest tightness. The electrocardiogram (ECG) showed narrow QRS tachycardia with a prolonged PR interval.

**Take-Home Message**

• In patients with slow pathway–dominant conduction in sinus rhythm, low-power ablation of the slow pathway should be performed under intravenous administration of isoproterenol, and the power can be increased if there are no signs of atrioventricular block.

## Differential Diagnosis

The differential diagnosis for a young patient presenting with narrow QRS tachycardia includes sinus tachycardia, atrial tachycardia, atrial flutter, junctional tachycardia, atrioventricular nodal re-entrant tachycardia (AVNRT), and atrioventricular re-entrant tachycardia.

## Investigations

**Figure 1:**
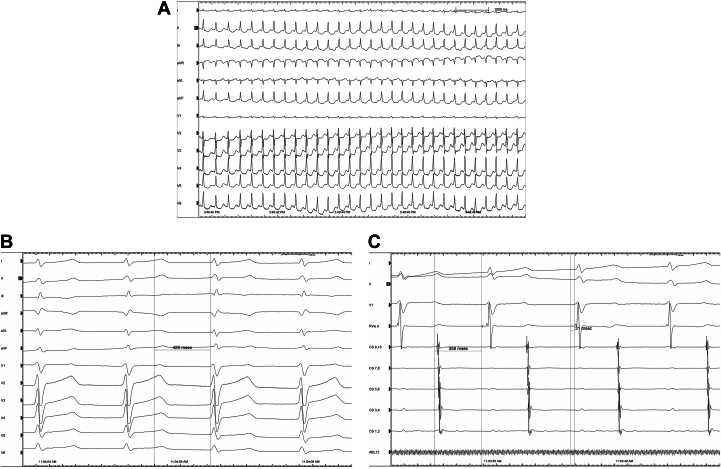
Preoperative Electrocardiographic Data (A) 12-lead electrocardiogram during palpitations. (B and C) Intracardiac electrogram variables (B: PR interval of 429 ms; C: AH interval of 358 ms and HV interval of 31 ms).

The ECG obtained from another hospital showed narrow QRS tachycardia (Figure 1A), and the patient was diagnosed with paroxysmal supraventricular tachycardia (PSVT).

**Figure 2:**
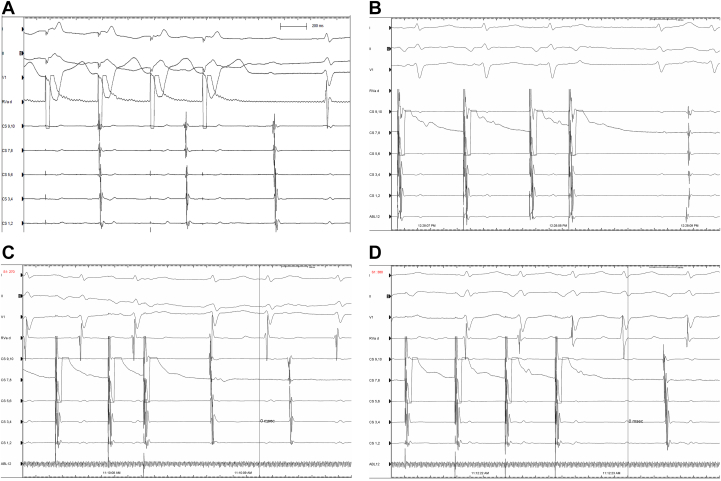
Electrophysiological Characteristics (A) Ventriculoatrial dissociation (ventricular S1S1: 400 ms stimulation). After intravenous isoproterenol, (B) jumping phenomenon (coronary sinus S1S2: 500/200 ms stimulation), (C) echo (coronary sinus S1S2: 500/270 ms stimulation), and (D) dual ventricular responses (coronary sinus S1S1: 300 ms stimulation) were seen.

A cardiac electrophysiology study performed in our hospital measured a PR interval of 429 ms (Figure 1B). Coronary sinus electrode, His bundle electrode and right ventricular electrode were placed via the right femoral vein. The AH interval was measured as 358 ms, and the HV interval as 31 ms (Figure 1C). Wenckebach ventriculoatrial conduction appeared during S1S1 ventricular stimulation, and the endocardial electrogram showed ventriculoatrial dissociation during S1S1 400 ms ventricular stimulation (Figure 2A). S1 showed Wenckebach conduction while coronary sinus S1S2 stimulation, which was performed again after intravenous isoproterenol, and atrioventricular conduction jumping was seen at 500/200 ms (Figure 2B). Echoes were seen during coronary sinus S1S2 500/270 ms stimulation (Figure 2C). Dual ventricular responses were seen during coronary sinus S1S1 300 ms stimulation after infusion of isoproterenol (ISO),^1^^,^^2^ which suggests that the atrial wave (A-wave) successively conducts through the fast pathway and slow pathway of the atrioventricular junction and activates the ventricle to form the ventricular wave (V-wave) (Figure 2D).

## Management

**Figure 3:**
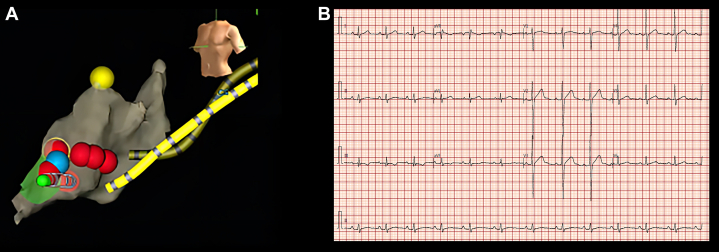
Ablation Targets and Postoperative Electrocardiogram (A) The ablation targets (red points) demonstrated on the left anterior oblique view of the three-dimensional geometry of the right atrium. (B) Postoperative 12-lead ECG. ECG = electrocardiogram.

The electrophysiology study was consistent with the dual atrioventricular nodal pathways with slow-fast AVNRT. The radiofrequency ablation target was located above the coronary sinus ostium (Figure 3A). After successful ablation of the slow pathway, the jumping and echo phenomenon of the atrioventricular node disappeared. The atrial impulses were conducted through the fast pathway, and the PR interval on ECG returned to normal (Figure 3B).

## Discussion

AVNRT is the most common cause of PSVT in clinical practice. It is classified based on different anterograde pathways into slow-fast, fast-slow, and slow-slow types. The slow-fast type accounts for 80% to 90% of cases; the characteristics of this tachycardia are anterograde slow pathway conduction and retrograde fast pathway conduction (leading His bundle A-wave). The AH interval is typically more than 200 ms and is significantly larger than the HA interval. On 12-lead ECG, this is usually shown as a narrow QRS tachycardia, with the P-wave usually hidden in the QRS wave or located at the end of the QRS wave, manifesting pseudo-R′ in lead V_1_ and pseudo–S-wave in leads II, III, and aVF, with an RP′ interval typically <70 ms; the ECG after the termination of tachycardia often shows a normal PR interval, suggesting that the impulses from the sinus node conduct through the fast pathway to activate the ventricle.

**Figure 4:**
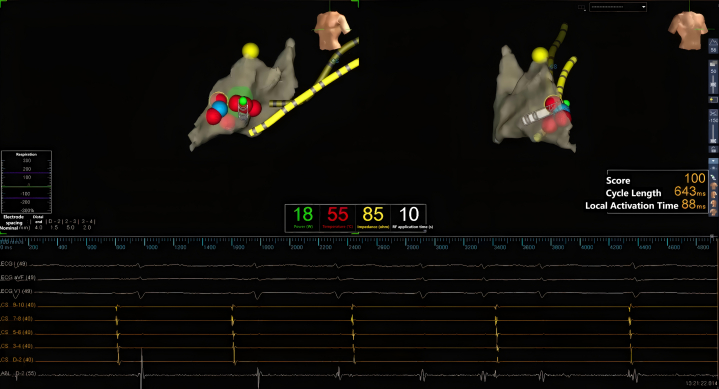
Radiofrequency Ablation Induced a Junctional Rhythm During the Procedure

In the current case, the patient's ECG manifested as a tachycardia with prolonged PR interval. The electrophysiology study indicated the existence of dual atrioventricular nodal pathways. The PR interval returned to the normal range after intravenous ISO, which indicated that at baseline, the patient had slow pathway–dominant conduction, resulting in a long PR interval on ECG. This phenomenon is relatively rare clinically. If the conduction function is poor, there is a risk of atrioventricular block after ablation of the slow pathway, so the ablation should be performed with caution. However, the patient's ECG showed a normal PR interval after the intravenous infusion of ISO, which indicated that the conduction function of the fast pathway was normal. Therefore, low-power ablation was initiated after intravenous ISO infusion firstly (Figure 4), and the power was increased to 35 W, as there were no signs of atrioventricular block. After successful ablation, the ECG showed normal PR interval, which confirmed that the functioning of the atrioventricular node fast pathway was restored.

Supraventricular impulses consistently conducted through the slow pathway to the ventricle, and the ECG showed first-degree atrioventricular block with long PR interval, which is very rare in clinical practice. The electrophysiological characteristics of dual atrioventricular nodal pathways are that the fast pathway has a swift rapid conduction velocity with a longer refractory period, while the slow pathway has a slow conduction velocity but a shorter refractory period. In this condition, the supraventricular electrical signal is easier to encounter the refractory period of the fast pathway, which leads to the functional conduction block and propagates impulse travels over the slow pathway that manifest as a prolonged PR interval on ECG. Furthermore, the slow pathway anterograde conduction also produces a concealed conduction to the fast pathway, which causes the fast pathway continuous functional conduction block, that is, the occurrence of the linking phenomenon between the fast and slow pathway.^3^ The above phenomenon may be associated with the inhibited conduction function of the fast pathway, which is due to the increased vagal tone or the electrical tension of the slow pathway.^3^

From the above characteristics, it can be inferred that there is a high risk of atrioventricular block during slow pathway ablation that operators should be aware of. Generally, we recommend starting at low-power radiofrequency ablation while closely monitoring atrioventricular conduction function, stopping the procedure immediately if there are any signs of atrioventricular block. In our case, the PR interval returned to normal after infusion of ISO, which indicated normal functioning of the fast pathway, so the slow pathway ablation was performed successfully and with low risk. After the procedure, the patient's sinus node impulses were conducted through the fast pathway to the ventricle, accompanied with a normalization of the PR interval.

## Conclusions

Our case demonstrated that AVNRT with dominant conduction of the slow pathway can be eliminated successfully and safely through ablation of the slow pathway, and that atrioventricular block can be avoided. Operators should ensure that the fast pathway is provided with normal conduction function.

**Visual Summary:**
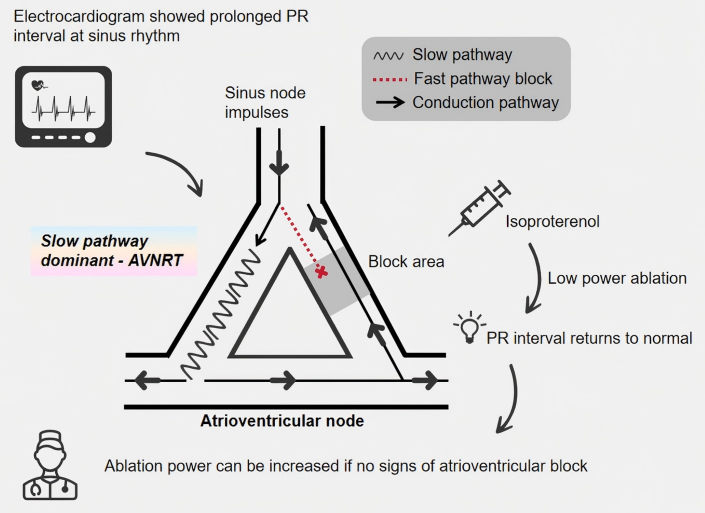
XXX AVNRT = atrioventricular nodal re-entrant tachycardia.

## Funding Support and Author Disclosures

The authors have reported that they have no relationships relevant to the contents of this paper to disclose.
